# Impact of Ultrasound- and Microwave-Assisted Extraction on Bioactive Compounds and Biological Activities of *Jania rubens* and *Sargassum muticum*

**DOI:** 10.3390/md22120530

**Published:** 2024-11-25

**Authors:** Kahina Hamamouche, Zoubida Elhadj, Latifa Khattabi, Wafa Zahnit, Brahim Djemoui, Omar Kharoubi, Walid Boussebaa, Mouhamed Bouderballa, Mohammed EL Moustapha Kallouche, Sabry M. Attia, Sheikh F. Ahmad, Maria Atanassova, Mohammed Messaoudi

**Affiliations:** 1 Environmental Monitoring Network Laboratory, Department of Biology, Faculty of Natural and Life Sciences, Ahmed Ben Bella University, Oran 31000, Algeria; 2 Laboratory for Environmental and Materials Sciences Studies, Ahmed Ben Bella University, Oran 31000, Algeria; 3 Biotechnology Research Center-C.R.B.T, Constantine 25017, Algeria; 4 Department of Chemistry, Faculty of Sciences, University of Ferhat ABBAS Setif 1, El Bez 19000, Algeria; 5 Laboratory of Excellence in Applied Organic Synthesis, Faculty of Exact and Applied Sciences, Ahmed Ben Bella University, Oran 31000, Algeria; 6 Laboratory of Experimental Biotoxicology, Biodepollution and Phytoremediation, Faculty of Natural and Life Sciences, Ahmed Ben Bella University, Oran 31000, Algeria; 7 Scientific and Technical Research Center in Physico-Chemical Analysis CRAPC BP384, Bou-Ismail 42004, Algeria; 8 Environmental Monitoring Network Laboratory, Faculty of Natural and Life Sciences, Ahmed Ben Bella University, Oran 31000, Algeria; 9 Department of Pharmacology and Toxicology, College of Pharmacy, King Saud University, Riyadh 11451, Saudi Arabia; 10 Scientific Consulting, Chemical Engineering, University of Chemical Technology and Metallurgy, 1734 Sofia, Bulgaria; 11 Nuclear Research Centre of Birine, Ain Oussera 17200, Algeria

**Keywords:** *Jania rubens*, *Sargassum muticum*, ultrasound assisted extraction, microwave-assisted extraction, LC-MS analysis, bioactive compounds, biological activities

## Abstract

This study represents the first investigation into the ultrasonic and microwave extraction of bioactive metabolites from *Jania rubens (J. rubens)* (red seaweed) and *Sargassum. muticum* (*S. muticum*) (brown seaweed), with a focus on their biological activities. The research compares ultrasound-assisted extraction (UAE) with microwave-assisted extraction (MAE) utilizing a hydromethanolic solvent to evaluate their effects on these seaweeds’ bioactive compounds and biological activities. The assessment included a series of antioxidant essays: DPPH, ABTS, phenanthroline, and total antioxidant capacity, followed by enzyme inhibition activities: alpha-amylase and urease. Results revealed significant proportions of phenolic compounds, ranging from 48.31 ± 0.32 to 74.42 ± 0.80 μg GAE/mg, depending on the extraction method. The extracts demonstrated a high antioxidant activity, with IC_50_ values ranging from 26.58 ± 0.39 to 87.55 ± 0.69 μg/mL. Notably, the MAE extract of *S. muticum* showed a value of 48.11 ± 2.75 μg/mL for alpha-amylase inhibition, which is strictly superior to the reference acarbose with an IC_50_ equal to 3431.01 μg/mL. UPLC-ESI-MS/MS analysis identified 14 bioactive compounds. The proportion of riboflavin with MAE was 70.58% and 59.11% for *J. rubens* and *S. muticum* fractions, respectively. These findings underscore the critical influence of extraction technique selection on bioactive compounds’ yield and efficiency, highlighting the potential of algal biomass as a sustainable alternative in various applications.

## 1. Introduction

Algae have been historically recognized as a sustainable resource for diverse applications, including animal feed, agriculture, and medicine, thus making significant contributions to the economy [1]. They play a crucial role in marine ecosystems as primary producers, serving as a food source for herbivorous organisms and providing habitats for various microorganisms [2]. Algae development and traits are affected by abiotic factors, including climate, salinity, pH, depth, light exposure, and ultraviolet radiation, as well as biotic factors, such as species, life stage, size, age, reproductive status, and herbivory intensity. In response to environmental stresses, macroalgae produce a variety of antioxidant compounds, particularly polyphenols, as a defense mechanism [3]. These organisms synthesize a broad spectrum of primary metabolites, including proteins, lipids, carbohydrates, and secondary metabolites such as polyphenols, phlorotannins, and carotenoids [4]. Macroalgae are classified into three primary groups based on their pigment composition: green algae (Chlorophyta), brown algae (Ochrophyta), and red algae (Rhodophyta) [2,3,5]. They exhibit diverse biometabolites, including essential amino acids, carotenoids, proteins, fatty acids, vitamins, dietary fibers, and minerals [3]. This bioresource holds extensive biotechnological potential and can be applied in pharmaceuticals, food and animal production, agriculture, and cosmetics [4,6]. Furthermore, algae are recognized for their various bioactivities, such as antioxidant, antimicrobial, anti-obesity, anticancer, antifungal, anti-inflammatory, antiallergic, hypoglycemic, cytotoxic, and antiviral properties [6,7].

The red alga *Jania rubens* (L.) Lamx. (formerly *Corallina rubens* L., family Corallinaceae, Rhodophyta) (*J. rubens)* [8] exhibits a broad geographical range, thriving in regions such as the Mediterranean and Black Seas, the northeastern Atlantic (from Norway to Morocco) and Indian Oceans, and the China Sea [9]. The multicellular thalli of *J. rubens* consists of a filamentous structure that forms the thallus’s foundation [10]. This species has been extensively studied for its various properties, including antitumor activity [8], antimicrobial potential [9], and cytotoxic activity [11]. Notably, it demonstrates antibacterial capabilities against human pathogens [1] and has industrial applications as a thickener and gelling agent [12]. Furthermore, it exhibits hypoglycemic, fibrinolytic, and lipolytic activities [8]. The anticancer effects of *J. rubens* extracts have been documented in numerous studies; research into its cancer-fighting potential is ongoing [11,13].

On the other hand, *Sargassum muticum (S. muticum)*, commonly known as Japanese wireweed, is recognized as a highly invasive brown seaweed, often classified as the most invasive species in certain contexts [14]. It thrives along the coastlines of the British Isles, mainland Europe, and North America [15]. *S. muticum* has been used in traditional medicine in Europe and the Americas for many years [16]. Its complex morphology resembles higher plants, characterized by a basal disc and pedicellate air vesicles [17]. This species is naturally abundant in antioxidant compounds and exhibits a range of biological activities, including antioxidant, antibacterial, and anti-inflammatory effects [18]. Additionally, it shows therapeutic potential [19] and contributes to bioremediation efforts [20] as well as various industrial applications [21]. The effectiveness of algal bioactive compounds against urease-positive infections has garnered significant research attention [22]. The growing interest in these algae’s bioactive compounds, along with their potential applications in the food, pharmaceutical, and nutraceutical industries, underscores the importance of this research area [23].

Recent technological breakthroughs have created novel extraction techniques, including ultrasound-assisted extraction (UAE) and microwave-assisted extraction (MAE) [24], which enhance the production of high-quality bioactive compounds. Ultrasound-assisted extraction employs frequencies ranging from 20 kHz to 10 MHz, inducing cavitation bubbles within the extraction medium. This phenomenon disrupts cellular structures, thereby facilitating the transfer of valuable compounds into the solvent [6,25]. In contrast, microwave-assisted extraction uses electromagnetic waves with frequencies from 300 MHz to 300 GHz, generating heat through ionic conduction and dipolar movement in the presence of polar solvents [26].

Our study seeks to explore the biological activities of two Algerian algae, *J. rubens* and *S. muticum*, using innovative extraction methods: ultrasonic and microwave techniques. We aim to identify the profiles of their bioactive compounds through a comprehensive approach. To thoroughly assess their antioxidant properties, we used four different tests: DPPH, ABTS, phenanthroline, and total antioxidant capacity. We also evaluated the alpha-amylase and urease inhibitory activities of these natural sources. The findings highlight their potential health benefits and contribute to a deeper understanding of their bioactive compounds. Additionally, this research underscores the importance of new extraction techniques and the significance of a green chemistry approach that prioritizes environmental sustainability.

## 2. Results

### 2.1. Extraction Yield

The extraction yield was quantified as follows in Table 1. Precisely, the extraction yields for *J. rubens* were measured at 6.84 ± 0.60 and 6.97 ± 0.10 for the UAE and MAE methods, respectively. Similarly, the extraction yields for *S. muticum* were determined to be 23.66 ± 0.10 and 24.84 ± 0.11 for the MAE and UAE methods, respectively. Interestingly, the UAE method demonstrated higher efficiency for extracting *S. muticum*, whereas the MAE method was more effective for *J. rubens* extraction. Notably, the difference between the yields obtained by these methods was minimal.

### 2.2. Scanning Electron Microscopy (SEM) & Energy Dispersive X-Ray Spectroscopy (EDX)

**Figure 1:**
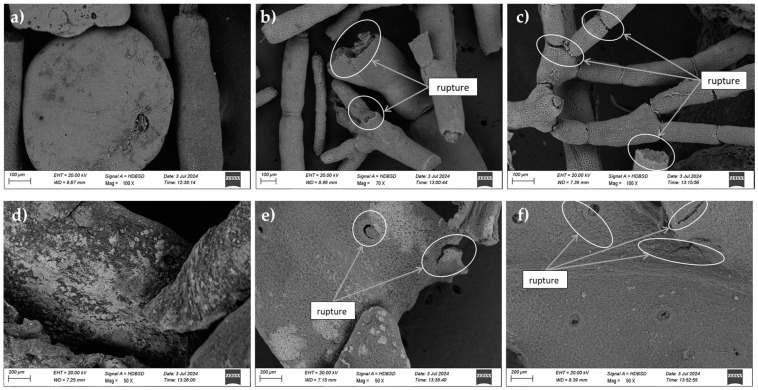
SEM images of *J. rubens* and *S. muticum*: (**a**) *J. rubens* before extraction; (**b**) *J. rubens* after MAE treatment; (**c**) *J. rubens* after UAE treatment; (**d**) *S. muticum* before extraction; (**e**) *S. muticum* after MAE treatment; (**f**) *S. muticum* after UAE treatment.

The SEM image of *J. rubens* reveals that the untreated biomass has a surface matrix characterized by closed cells that appear rough, dark, and heterogeneous, with intact cell walls showing no signs of rupture in Figure 1a. After MAE, Figure 1b illustrates the damaging effects of the waves on the cellular morphology, resulting in visible damage. Figure 1c indicates that it is also damaged after UAE treatment. On the other hand, before treatment, *S. muticum* displays a rough, flaky surface morphology with interconnected aggregates forming a mosaic-like pattern in Figure 1d. Following microwave treatment, Figure 1e shows notable alterations in the surface structure, while Figure 1f highlights significant morphological changes after ultrasound treatment.

### 2.3. Energy-Dispersive X-Ray Spectroscopy

**Figure 2:**
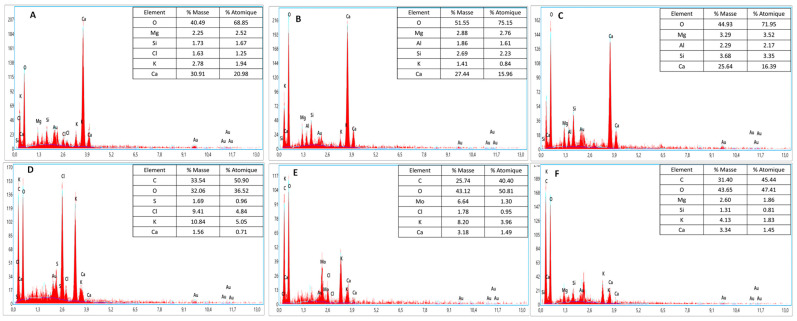
EDX analysis results: (**A**) *J. rubens* before treatment; (**B**) *J. rubens* after MA; (**C**) *J. rubens* after UAE; (**D**) *S. muticum* before treatment; (**E**) *S. muticum* after MAE; (**F**) *S. muticum* after UAE.

Before treatment, the biomass composition of *J. rubens* showed oxygen at 40.49%, followed by potassium and, subsequently, calcium, magnesium, chlorine, and silicon, as shown in Figure 2A. After UAE, the oxygen weight percentage increased to 44.93%, rising further to 51.55% after MAE. This indicates that the extraction techniques significantly influence the elemental composition of the biomass. Notably, aluminum was undetectable before treatment but appeared afterwards, likely due to being previously hidden. Furthermore, chlorine and calcium were only identified in the X-ray spectrum after the UAE treatment, indicating that the UAE technique influenced these elements.

The X-Ray spectrum mapping for *S. muticum* revealed a high carbon content of 33.54% of the total mass, followed by oxygen at 32.06%, potassium at 10.84%, chloride at 9.41%, sulfur at 1.69%, and calcium at 1.56%. These percentages reflect the elemental composition before MAE or UAE treatment, as illustrated in Figure 2D–F. After MAE treatment, the carbon mass percentage decreased slightly to 31.40% and 25.74%. Conversely, the oxygen percentage increased to 43.12% and 43.65% after MAE, as shown in Figure 2E. Interestingly, sulfur was only detected following MAE, indicating chlorine was likely observed before this treatment. Overall, the X-ray spectrum profile of *S. muticum* confirms that the extraction technique significantly alters the surface elemental composition.

### 2.4. Determination of the Bioactive Molecules by Liquid Chromatography-Mass Spectrometry (LC-MS-MS)

**Figure 3:**
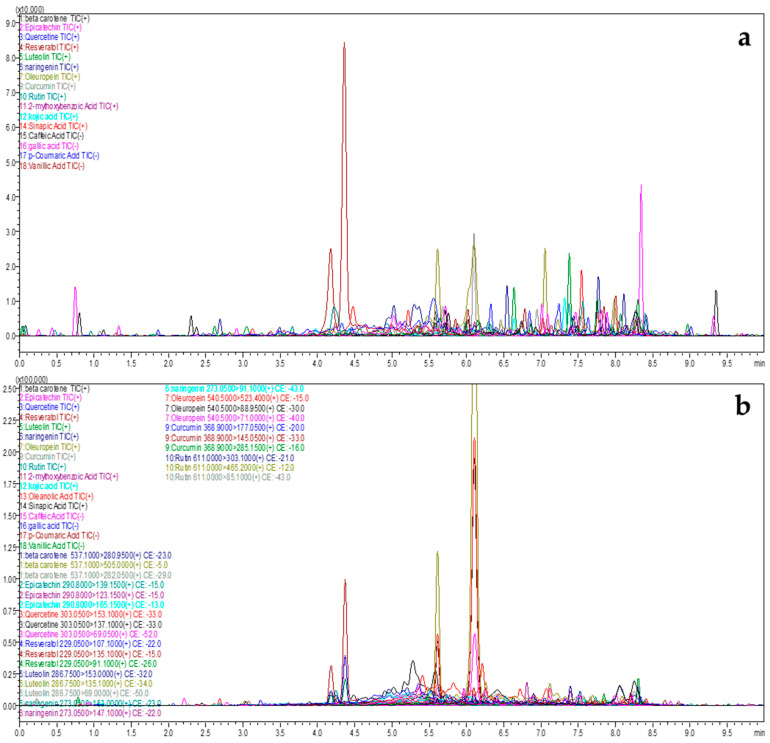
Bioactive components profile of *J. rubens* determined by UPLC-ESI-MS-MS. (**a**) MAE *J. rubens*; (**b**) UAE *J. rubens*.

**Figure 4:**
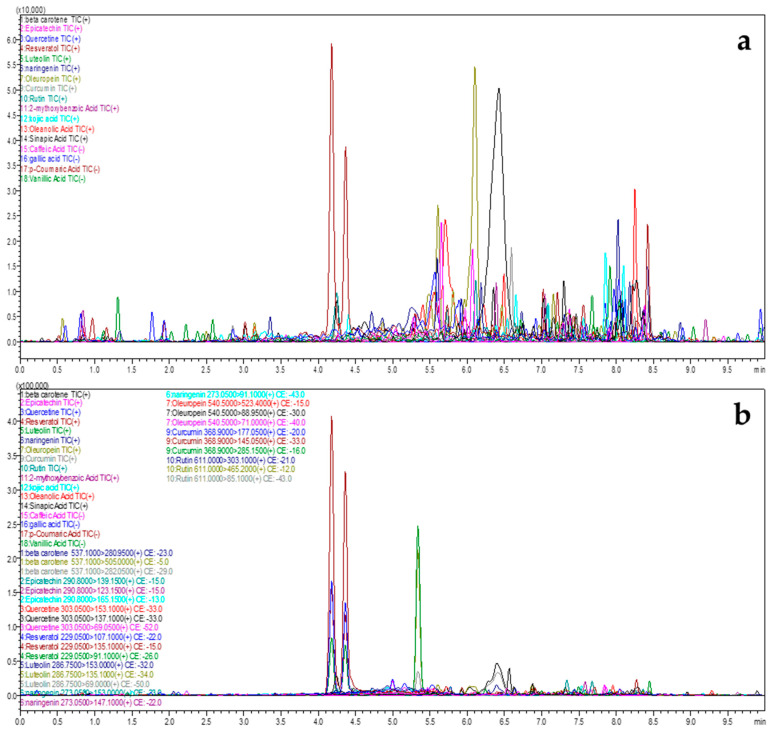
UPLC-ESI-MS-MS-determined bioactive components profile of *S. muticum*. (**a**) MAE *S. muticum*; (**b**) UAE *S. muticum*.

The fractions of *J. rubens* and *S. muticum* were submitted to UPLC/MS-MS analysis, which led to the identification and characterization of 14 bioactive compounds. This was accomplished by comparing the duration of their retention with that of established benchmarks. The outcomes of the compounds identified in the *J. rubens* and *S. muticum* fractions using UPLC/MS-MS are presented in Table 2, Figure 3, and Table 3, Figure 4, respectively.

**Figure 5:**
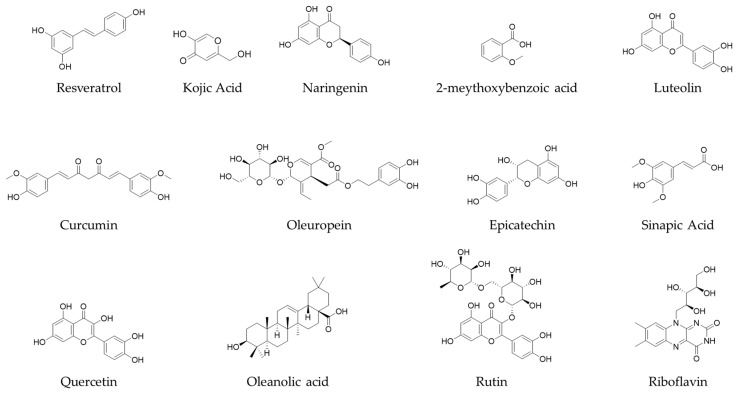
Chemical structure of identified seaweed bioactive compounds.

The UPLC-ESI-MS/MS study of extracts from *J. rubens* and *S. muticum* indicated the presence of diverse bioactive chemicals in all the extracts. The compounds mentioned are phenolic acids, specifically 2-methoxybenzoic acid, kojic acid, oleanolic acid, and sinapic acid. Flavonoids were also detected, including epicatechin, quercetin, resveratrol, luteolin, naringenin, oleuropein, and rutin (Figure 5).

The extracted bioactive components were identified through a qualitative study of *J. rubens* and *S. muticum* extracts using liquid chromatography (LC) coupled with tandem mass spectrometry (MS2) in positive and negative ionization modes. From this viewpoint, we aimed to identify the polyphenolic profiling of a recently popularized set of environmentally friendly methods that have gained significant attention in scientific research: microwave-assisted extraction (MAE) and ultrasonic-assisted extraction (UAE). The results obtained for each extract showed a distinct chemical profile, dictated by the specific type of algae used and the method of extraction used. The percentages of each compound, a variable influenced by the extraction method and the type of algae studied, indicate the complex nature of the chemical profile. The phenolic acids identified included 2-methoxybenzoic acid, sinapic acid, p-coumaric acid, and kojic acid, among others, adding to the complexity of the research.

The results of the qualitative analysis by LC-MS-MS of the different extracts obtained using various extraction methods from two algae, *J. rubens* and *S. muticum*, are shown in Table 2 and Table 3. The bioactive components found were the same for both algae, but a difference was observed in terms of kojic acid. It was not detected in the MAE *J. rubens*, but was found in the UAE *J. rubens*. Additionally, kojic acid was not detected in the UAE *S. muticum*. Based on these data, the composition of the studied algae is reasonably similar, with some variations.

### 2.5. Phenolic and Flavonoid Content

As depicted in Table 4, the phenolic content of *J. rubens* extracts was as from 48.31 ± 0.32 to 71.83 ± 0.96 μg GAE/mg, while *S. muticum* extracts exhibited a range of 68.5 ± 0.96 to 74.42 ± 0.80 μg GAE/mg. The flavonoid content was notably higher in *J. rubens* when extracted using the MAE method, yielding 38.04 ± 0.98 μg QE/mg compared to 31.38 ± 0.73 μg QE/mg obtained through the UAE method. Similarly, for *S. muticum*, the flavonoid content was more significant with MAE, measuring 58.47 ± 0.24 μg QE/mg, in contrast to 42.65 ± 0.36 μg QE/mg with UAE. Tannin levels were higher in *S. muticum* extracts using the UAE method, at 133.67 ± 1 μg EqC/mg, compared to 107.52 ± 2.6 μg EqC/mg with MAE. In the case of *J. rubens*, the tannin content was greater with MAE, recorded at 64.19 ± 0.76 μg EqC/mg, compared to 52.65 ± 1.33 μg EqC/mg with UAE. These findings indicate that the results obtained from the MAE method for *J. rubens* were superior to those from UAE, whereas for *S. muticum*, UAE demonstrated higher yields, highlighting the differential extraction efficiencies of these methods for the two algal species.

### 2.6. Antioxidant Activities

The antioxidant activity of all extracts was evaluated using spectrophotometric methods with a microplate reader, and the results of all experiments are displayed in Table 5.

#### 2.6.1. Diphenyl 1-Picrylhydrazyle (DPPH) Scavenging Activity

In the current study, the algae extracts, *J. rubens* and *S. muticum*, displayed dose-dependent anti-radical properties. As presented in Table 5, *J. rubens* extracted by MAE and UAE showed noteworthy IC_50_ values of 57.13 ± 0.75 μg/mL and 53.97 ± 0.39 μg/mL, respectively. For *S. muticum*, the IC_50_ values were 87.55 ± 0.69 μg/mL for MAE and 32.67 ± 0.43 μg/mL for UAE. These values were higher than those of the reference compounds, which ranged from 2.03 ± 0.01 μg/mL for BHA to 6.28 ± 0.14 μg/mL for vitamin C (Table 5). The extract from *S. muticum* obtained via UAE exhibited the highest potency in this assay, with an IC_50_ value of 32.67 ± 0.43 μg/mL, indicating that the UAE method had a more significant effect on both seaweeds.

#### 2.6.2. Azion-Bis 3-Ethylbenzothizoline-6-Sulfonic Acid (ABTS) Scavenging Activity

According to Table 5, inhibition percentages were proportionally increased with increased concentrations, reaching 90.43 ± 0.45% for UAE *J. rubens*, 90.28 ± 0.52% for UAE *S. muticum*, 89.24 ± 0.37% for MAE *J. rubens*, and 88.70 ± 0.26% for UAE *S. muticum*. Similarly to the DPPH assay, the strongest extract was UAE- *J. rubens*, exhibiting the best IC_50_ value (26.58 ± 0.39 μg/mL), followed by MAE *J. rubens* (31.38 ± 0.01 μg/mL). However, for *S. muticum*, the value was 44.56 ± 0.78 μg/mL with UAE and 48.18 ± 0.45 μg/mL with MAE. These IC_50_ values are less significant than those of the reference compounds (1.73 ± 0.23 μg/mL, 2.09 ± 0.07 μg/mL and 27.63 ± 0.58 μg/mL for BHT, BHA, and vitamin C, respectively).

#### 2.6.3. Phenanthroline Activity

The ability of *J. rubens* and *S. muticum* extracts to reduce Fe^3+^ to Fe^2+^ in the presence of phenanthroline reagent was expressed as the A0.5 in Table 5. The absorbance of phenanthroline activity decreased as the concentration decreased in all extracts; the highest values of inhibitory percentage ranged from (70.236 ± 0.59%) for MAE *S. muticum* to (28.194 ± 0.60%) in UAE *S. muticum.* On the other hand, the value of A0.5 was higher with MAE *S. muticum* extract (61.74 ± 0.86 μg/mL), followed by UAE *J. rubens* (79.80 ± 0.68 μg/mL), MAE *J. rubens* (87.98 ± 0.55 μg/mL), and UAE *S. muticum* (97.41 ± 0.58 μg/mL). In this study, the UAE method gave a better result for *J. rubens* against MAE for *S. muticum*.

#### 2.6.4. Total Antioxidant Capacity

The results of this assay, expressed as absorbance at A0.5, are detailed in Table 5. The data revealed that the A 0.5 value for *J. rubens* extracted via MAE was 192.42 ± 0.69 μg/mL, which was lower (indicating greater activity) compared to the UAE extraction of *J. rubens*, which yielded an A 0.5 value of 249.98 ± 0.51 μg/mL. Similarly, for *S. muticum*, the A 0.5 value for the MAE method was 404.55 ± 0.97 μg/mL, which was significantly lower than that obtained through UAE (730.56 ± 0.96 μg/mL). The MAE extracts of *J. rubens* and *S. muticum* exhibited more favorable A 0.5 results than their UAE counterparts. It is important to note that these A 0.5 values exceeded the standards, which are 18.94 ± 0.38 μg/mL for BHA and 37.91 ± 0.24 μg/mL for vitamin C.

### 2.7. Inhibition of Enzymatic Activity

#### 2.7.1. Inhibition of Alpha-Amylase Activity

Table 6 indicates that among several extracts obtained through both extraction procedures, only the MAE extract of *S. muticum* showed significant α-amylase inhibitory activity, with an IC_50_ value of 48.11 ± 2.75 μg/mL. In the other three extracts, no α-amylase inhibition activity was recorded.

#### 2.7.2. Inhibition of Urease Activity

The results (Table 6) indicated that the MAE and UAE extracts of *S. muticum* yielded similar outcomes, with values ˂ 3.125(μg/mL)., which is strictly superior to the standard potential. For *J. rubens*, the UAE extract exhibited higher activity, with an IC_50_ equal to 75.24 ± 3.36 μg/mL, compared to the MAE extract, which had an IC_50_ value of 134.79 ± 1.35 μg/mL.

## 3. Discussion

Algae have become a significant resource in the scientific community. They are renowned for their abundance and renewability and for producing a diverse range of innovative bioactive chemicals with considerable promise for use in the pharmaceutical and cosmetics sectors [4]. However, the yield and quality of extraction from algal material represents a critical and sensitive stage in the research process, as these factors can substantially impact the results obtained [27]. The cellular structure of algae is complex, and the target compounds are difficult to extract with conventional extraction methods [25]; these limits give birth to innovative extraction processes such as MAE and UAE. These technologies will replace difficult extraction techniques that have higher energy consumption and high pollution characteristics [6,28].

Previous studies have examined the extraction yield of *J. rubens* using conventional extraction methods [5]. The UAE methodology resulted in a higher extraction yield *for J. rubens* than previous studies, which reported an average yield for conventional techniques. Conversely, the MAE method resulted in a reduced extraction yield for *J. rubens* [29]. Furthermore, the maceration method, which was employed to extricate Sargassum species, produced extraction efficiencies that varied significantly among studies when employing a variety of solvents. Our findings suggest that the UAE method outperformed the maceration technique in terms of yield. The ratio of material to solvent is a crucial factor in the UAE method, as a higher ratio enhances the contact area between the material and solvent, potentially leading to increased extraction efficiency [6,30]. Furthermore, innovative extraction methods are recognized as more efficient, sustainable, and environmentally friendly than traditional techniques [7].

### 3.1. Scanning Electronic Microscopy (SEM)

Scanning electron microscopy (SEM) was employed to evaluate the influence of processes for extraction on the cellular structures of the two algae species, as demonstrated in Figure 1 and Figure 2.

The SEM images demonstrated that the cell surface of the untreated *J. rubens* samples was intact, unaltered, and appeared smooth, featuring large, lobed structures (Figure 1a) and branched formations (Figure 1b). The morphological surface of *J. rubens* was altered by MAE, as demonstrated in Figure 1c,d. This confirms that the cells were substantially damaged, with a substantial destruction of cell walls. The enhanced yield is attributable to the fact that the majority of the cell walls were constricted and fractured. However, after undergoing ultrasound treatment, the images revealed increased porosity in the algal biomass, as shown in Figure 1e. In another work by Rodriguez-Jasso et al. [31] and Garcia-Vaquero et al. [32], the effect of UAE and MAE on the cell wall was confirmed.

### 3.2. Energy-Dispersive X-Ray Spectroscopy (EDX)

According to the analysis of the X-ray spectrum for *J. rubens*, the results in Figure 2 show that O, Mg, S, Cl, K and Ca are the major constituents of the macroalgal cell surface, whereas the results obtained by Parthiban et al., 2013 [33] showed that Fe is present in *J. rubens*. The element Al was not detected, which means that it was covered. After treatment with MAE or UAE treatment, Figure 2A and 2B, respectively, show that the concentrations of atomic elements increased for O and Mg and decreased for K and Ca. Al was detected, but Cl was not present in the X-ray spectrum. This means that Cl was affected by the MAE and UAE extraction methods.

The mapping X-ray spectrum for *S. muticum* in Figure 2 shows that, on the one hand, C, O, S, Cl, K and Ca are naturally present on the surface of this biomass; on the other hand, C and O are the most abundant elements. We noticed that the latter observation was similar to that presented in the work of Fawzy et al. [34]. Following treatment with MAE Figure 2E, the atomic values for C, Cl, and K diminished, but O experienced an increase, and S remained unchanged. Magnesium and silicon were undetectable owing to their sensitivity to the MAE. The effect of ultrasound on *S. muticum* is illustrated in Figure 2C, which shows a decrease in the elements C and K. The value of O and Ca increased with Mg and Si, meaning they were covered in natural biomass. The X-ray spectrum in this profile did not reveal the presence of Cl and S elements, suggesting that the ultrasound extraction process influenced their removal.

### 3.3. Analysis Conditions of the Liquid Chromatography-Mass Spectrometry (LC MS-MS)

The extracted bioactive components from *J. rubens* and *S. muticum* were identified using a qualitative analysis via UPLC-ESI-MS/MS Shimadzu 8040. This technique involved liquid chromatography and tandem mass spectrometry in both positive and negative ionization modes. From this viewpoint, we aimed to identify the polyphenolic profiling of a recently popularized set of environmentally friendly methods that have gained significant attention in scientific research: MAE and UAE. The results obtained for each extract demonstrated a distinct chemical profile, dictated by the specific type of algae used and the method of extraction used. The percentages of each compound are a variable influenced by the extraction method and the type of algae studied, indicating the complex nature of the chemical profile. The phenolic acids identified included 2-methoxybenzoic acid, sinapic acid, p-coumaric acid, and kojic acid, among others, adding to the complexity of the research.

As evidenced, the MAE extract of *J. rubens* is excessively rich in polyphenols, notably riboflavin and resveratrol, which account for the highest peak area proportions at 70.58% and 12.26%, respectively. Riboflavin, characterized by a peak area proportion of 31.95%, coexists with oleuropein, which is present at 49.27% in the UAE extract of *J. rubens*. Resveratrol and riboflavin are also abundant in both *S. muticum* extracts, with resveratrol levels ranging from 8.88% to 52.29% and riboflavin levels between 31.74% and 59.11%. The flavonoid content is minimal, as evidenced by quercetin, which accounts for 2.40% in both the *S. muticum* MAE and UAE extracts, and luteolin at 1.77% in the *J. rubens* MAE and 0.03% in the UAE. The S. *muticum* MAE and UAE fractions reveal low levels of sinapic acid. Benzoic acid was detected in all fractions ranging from 0.2% to 1.35%. Additional compounds including naringenin, quercetin, rutin, cinnamic acid, and beta-carotene were also identified. Based on these data, the compositions of the investigated algae show some differences. This suggests that their growth environment, extraction method, geographical location, and interactions with other organisms significantly influence their chemical profiles.

### 3.4. Phenolic Compounds

The total phenolic content of algae, specifically the phenolic compounds (phlorotannins) composed of phloroglucinol units, is often quantified using the spectrophotometric Folin–Ciocalteu method, a standard approach for assessing phenolic content in plants [25,35]. Previous studies have reported varying levels of phenolic content in *J. rubens* and *S. muticum* extracts using different extraction methods. For instance, methanolic extracts of *J. rubens* showed significant concentrations of phenolic and flavonoid chemicals, along with saponin, tannins, and alkaloids [36]. Another study reported a lower level of phenolic compounds in *J. rubens* methanolic extract [37]. In the case of *Sargassum* sp., the UAE method yielded a substantial quantity of phenolic compounds, which was influenced by extraction process variables such as ratio, time, and power [38]. In contrast, the maceration method yielded a lower phenolic content for *S. muticum* [39]. Furthermore, previous research has shown that the UAE method yields more phenolic content than the maceration method [23,40], and a comparison with other algae indicated that phenolic compounds were higher in the UAE method than in the MAE method [41]. Macroalgae are recognized for their richness in biologically active molecules, particularly polyphenols and dietary fiber [42]. Algal phenolic compounds exhibit diverse molecular weights and structures, yet share similar chemical properties, and seaweeds are also known for their health benefits due to the presence of fucoidans, carrageenans, terpenoids, polyunsaturated fatty acids, and sulphated polysaccharides [14,23].

### 3.5. DPPH and ABTS Activities

The antioxidant properties of seaweed are garnering heightened interest due to the association of oxidative stress with the development of severe diseases. Reactive oxygen species (ROS) are generated during metabolic processes and can induce oxidative damage to biomolecules, resulting in numerous chronic illnesses [5,27]. DPPH and ABTS assays, which assess the capacity of compounds to donate hydrogen or scavenge free radicals, have been commonly used to evaluate the antioxidant activity of both *J. rubens* and *S. muticum* using various extraction methods [37,39]. Our results showed higher antioxidant activity compared to previous studies, and it has been observed that methanolic and aqueous extracts of certain red seaweeds exhibit strong antioxidant effects in DPPH assays [24,43]. Additionally, it has been reported that *J. rubens* extract had the highest free radical scavenging activity (DPPH). Research has demonstrated the effective antioxidant properties of *S. muticum* extracts derived from green extraction methods [44] as well as a notable presence of phenolic chemicals with antioxidant benefits in extracts from the *Sargassum* genus [45]. While no studies have been found to extract phenolic compounds from *J. rubens* using UAE or MAE methods, the antioxidant activity of sulfated polysaccharides extracted from *J. rubens* using the UAE method has been assessed using the DPPH assay [46].

### 3.6. Phenanthroline Activity and Total Antioxidant Capacity

The assessment of antioxidant activities such as reducing power, phenanthroline assay, and total antioxidant capacity is grounded in the principle of electron transfer. The phenanthroline assay relies on the reaction between 1,10-phenanthroline and ferrous ions (Fe^2+^), resulting in the formation of a red-orange tri-phenanthroline complex that absorbs light at 508–510 nm (A510) and is used to quantify iron in diverse samples [47]. The reduction of Fe^3+^ is highly correlated with other antioxidant qualities and is often utilized as an indicator of electron-donating activity, which is a crucial mechanism of phenolic antioxidant action [48]. Previous studies have demonstrated the diverse antioxidant activities of red seaweeds, including *J. rubens*, Porphyravietnamensis, and Acanthophoraspicifera, as assessed by various methods such as DPPH, reducing power, total antioxidant capacity, ferrous ion chelating activity, and H_2_O_2_ scavenging activity, with varying inhibitory percentage results across different activities and extracts. Similarly, *S. muticum* and other macroalgae have been found to have substantial reducing power, highlighting the potential of phenolic compounds in macroalgae as sources of electron-donating molecules [39]. However, no studies have been found regarding the phenanthroline assay results for the studied algae. These findings underscore the importance of using multiple methods to evaluate antioxidant activity. The extraction techniques, UAE and MAE, are crucial for isolating phenolic substances. Our findings demonstrate that the UAE approach is proficient in collecting proton-donating compounds, whereas the MAE method is superior for extracting electron-donating compounds. Furthermore, the sensitivity of phenolic compounds to extraction conditions has been highlighted, consistent with the findings of previous studies on the influence of various parameter changes on *S. muticum* TPC and its antioxidant activity [32].

Many researchers have proven linear relationships between the antioxidant capacities of various seaweeds and their phenolic content, indicating that the polyphenols in the extracts retain their chemical affinity despite differences in extraction processes [49]. This emphasizes the significance of evaluating the extraction procedure and its impact on seaweed extracts’ biological functions and chemical composition.

### 3.7. Inhibition of Alpha-Amylase Activity

Diabetes is a metabolic disease characterized by persistent hyperglycemia. There are numerous medicinal techniques for the treatment of type 2 diabetes [49,50]. Currently, several drug therapies, including hypoglycemic agents, insulin, and its analogues, are used to manage type 2 diabetes and reduce the risk of complications. Among natural resources, algae seems to be a promising alternative for diabetes treatment [51]. Our results demonstrated that the MAE extract of *S. muticum* showed a notable inhibition level of IC_50_ 48.11 ± 2.75 μg/mL, which is significantly higher than the reference acarbose, with IC_50_ equal to 3431.01 ± 2.72 μg/mL. Conversely, the UAE extracts of *S. muticum* and *J. rubens* show no alpha-amylase inhibitory activity. According to the result of a study, the alpha-amylase inhibition obtained with a maceration technique followed by ultrasound treatment of *J. rubens* presented a value of 2349.16 (μg/mL) [50].

Another study on *S. muticum* indicated deficient α-amylase inhibition (IC_50_ > 5000 μg/mL); however, the extract enriched with phlorotannins was particularly effective against α-glucosidase, with an IC_50_ of 27.06 (μg/mL) [51].

The findings indicate that the spatial and temporal variability in the bioactive compound composition of the collected seaweed is predominantly influenced by local environmental factors, including nutrient availability, suspended particulates, and, consequently, light availability, which significantly affect the chemical profile of seaweed biomass. The factors influencing the makeup of bioactive chemicals in seaweed communities are now up for debate. Consequently, the findings in this work is valuable for comprehending ecosystem dynamics and for the ecological evaluation and modeling of seaweed. Further research is essential, especially in different ecoregions, to validate the findings of this study and derive more comprehensive conclusions on the five examined seaweed groups. Future research should focus on the impact of environmental factors on the seaweed communities under more investigation and comprehension. Local site-specific factors influence the composition of seaweed bioactive compounds, particularly in terms of short-term temporal dynamics, albeit primary agents limit them. Consequently, diverse scales of environmental and structural elements must be incorporated into research.

### 3.8. Urease Inhibition Activity

The hydrolysis of urea by urease is responsible for the excessive production of ammonia. Thus, it leads to a significant increase in pH and creates a conducive environment for microorganism proliferation [52,53].

Diseases associated with urease activity, such as gastric ulcers, gastritis, and stomach cancer, are notably linked to *Helicobacter pylori* (*H. pylori*), a well-known human pathogen that colonizes the stomach due to increased pH levels [54,55].

The urease inhibition activity of the MAE and UAE extracts of *S. muticum* was particularly promising, with an IC_50_ of less than 3.125 μg/mL, significantly surpassing the standard. The inhibition rate of *S. muticum* through maceration was 35.65% ± 0.36. Other studies indicated that *Padina boergesenii* also shows potential urease inhibition [56]. According to our knowledge, there are no prior reports in the literature related to urease inhibition activity of *J. rubens* and *S. muticum.*

## 4. Materials and Methods

### 4.1. Sample Collection and Preparation

Our study was based on two selected algae, *J. rubens* and *S. muticum*, identified and authenticated by Dr. W. Khitri in the Department of Pharmacy at the Faculty of Medicine at the University of Ahmed Ben Bella Oran 1. These algae were harvested from the coasts of Ain Franine and Oran, located northwest of Algeria. The Krichtel forest borders this site from the east, the Kanastel forest from the west, the Mountain of Lions from the south, and the Mediterranean Sea from the north. These plants were collected and carefully cleaned with distilled water to remove necrotic parts and epiphytes and dried in the dark at room temperature. The dried samples were ground to fine powders and stored in airtight bags at 4 °C for later use.

### 4.2. Extraction Procedure

#### 4.2.1. Ultrasound-Assisted Extraction (UAE)

Ultrasound-assisted extraction was performed according to the following procedure, with some modifications [25]: ten grams each of *J. rubens* and *S. muticum* powders were mixed with 100 mL of methanol solvent (80%) and water (20%), and underwent an ultrasound-assisted extraction using a Bushi apparatus at 25 °C and 90% of sonication amplitude for 15 min for each sample.

#### 4.2.2. Microwave-Assisted Extraction (MAE)

For this procedure, we undertook the following extraction protocol with some modifications: 10 g of each precedent powder was mixed with 100 mL of methanol solvent (80%) and underwent microwave-assisted extraction using a Bushi apparatus at 25 °C and at a rate of 90% for 15 min [22].

The different extractions were repeated in triplicate, and the mixtures of each extraction were filtered, combined, and concentrated by a rotavapor (Bushi) at 38 °C. The hydro-methanolic extracts of *J. rubens* (UAE *J. rubens*, MAE *J. rubens*) and *S. muticum* (UAE *S. muticum*, MAE *S. muticum*) were weighted and stored at 4 °C for further analysis. Yield of extraction = (extract weight/initial powder weight) × 100.

### 4.3. Scanning Electronic Microscopy (SEM)

The surface morphology of the selected algae was analyzed using scanning electron microscopy (SEM EVO 15, ZESS. JOEL, campus, Hannover, Germany) coupled with energy-dispersive X-ray spectroscopy to determine the components of these dry seaweeds before and after treatment.

### 4.4. Energy-Dispersive X-Ray Spectroscopy (EDX)

The purpose from this analysis (Energy-dispersive X-ray spectroscopy, (Quanta FEG 250, Shizuoka, Japan) is to provide the elemental curve as output. This analytical technique is generally used in conjunction with SEM. EDX mainly detects X-rays emitted by the sample during the electron beam bombardment process to characterize the sample’s elemental composition of interest. Quantitative results can be obtained from the relative counts of X-rays at the characteristic energy levels of the sample constituents.

### 4.5. Analysis Conditions of the Liquid Chromatography-Mass Spectrometry (LC-MS-MS)

The phytochemical analysis was performed by Ultra-Performance Liquid Chromatography-Electrospray Ionization-Tandem Mass Spectrometry (UPLC-ESI-MS/MS, Shimadzu 8040, Kyoto, Japan). Using a UPLC-ESI-MS-MS Shimadzu 8040 Ultrahigh sensitivity with UFMS technology equipped through a binary bomb Nexera XR LC-20AD, the quantification of various phytochemical compounds in *J. rubens* and *S. muticum* fractions was carried out. The separation was accomplished using an Ultra-force C18 column (I.D, 150 mm 4,6 mm, 3 m particle size; Restek, Bellefonte, PA, USA). The chromatographic separation was performed using water and formic acid at 0.1% grade LC-MS as phase A, and methanol grade LC-MS as phase B. The following gradient elution program was used: 98% A (0,1min to 1 min), 45% A (1 min to 2.5 min), 5% A (2.5 min to 7 min), 0% A (7 min to 9 min), and 98% A (9 min to 12 min). The sampling rate was 0.2 mL·min^−1^, the injection volume was 5 μL through a Millex-LCR (PTFE) filter with a 0.22 mm pore size, and the column temperature was set at 30 °C. The ESI conditions used in the LC-MS-MS were as follows: 230 KPs of CID gas; −6.00 Kv conversion dynode; 350 °C interface temperature; 250 °C temperature DL; 3.00 L·min^−1^ gas flow; 400 °C thermal block; and 10.00 L/min gas flow. A mass spectrometer detected negative and positive ions in MRM mode (multiple reaction monitoring).

### 4.6. Phenolic Content Determination

The phenolic content of the four extracts was determined using the Folin–Ciocalteau reagent (FCR). In a microplate well, 100 μL of FCR was added to 20 μL of each extract or methanol for the blank. After 7 min of incubation, 80 μL of carbonate of sodium (CaCO_3_) was added to the wells. The microplate of the reaction mixture was incubated in the dark for 2 h, and the absorbance was read at 760 nm using a microplate reader [57]. The phenolic content of each extract was expressed as μg of gallic acid equivalent (μg GAE/mg of extract).

### 4.7. Flavonoid Content Determination

The flavonoid content of the different extracts was determined based on the formation of a complex between aluminum trichloride and flavonoids. The mixture reaction contained 100 μL of each extract and 100 μL of 2% aluminum chloride (AlCl_3_ in ethanol). The mixture was incubated for 15 min and read at 415 nm using a microplate reader. Quercetin from Sigma Aldrich (St. Louis, MO, USA) was used as a standard, and a calibration curve was plotted in a concentration range of 0 to 250 μg/mL. All analyses were performed in triplicate, and flavonoid content was expressed as μg QE/mg of extract.

### 4.8. Determination of Total Condensed Tannins Content

The TCTC was determined using the vanillin assay [58]. To 10 μL of sample, 160 μL of vanillin/methanol solution (4%, *w*/*v*) was added and homogenized. Then, 80 μL of concentrated HCl was added and left at room temperature for around 20 min. The absorbance was measured against the blank at 500 nm. The TCTC was calculated from the linear regression equation of the standard curve of catechin (y = 0.004x − 0.038, R2 = 0.994) and expressed as catechin equivalents micrograms per milligram of extract (μg CE/mg Extract).

### 4.9. Antioxidants Activities

#### 4.9.1. Diphenyl 1-Picrylhydrazyle (DPPH) Activity

The free radical scavenging assay of the extracts was assessed using the 1,1-diphenyl-2-picrylhydrazyl (DPPH) radical scavenging method, originally proposed by [52], with minor modifications. Initially, mixtures consisting of 40 μL of samples or standards (BHT and BHA) at various concentrations (ranging from 0 to 800 μg/mL), or methanol for the negative control, and 160 μL of DPPH solution (4% in methanol) were prepared in microplate wells and incubated in darkness for 20 min. Subsequently, the absorbance was measured at 517 nm using a microplate reader. This assay was conducted in triplicate. The percentage of inhibition was calculated using the following formula:

I% = [(Abs_s_ − Abs_c_)/Abs_c_)] × 100

where Abs_s_ is the absorbance of the sample, and Abs_c_ is the absorbance of the negative control.

The IC_50_ was calculated from three successive points of percentage inhibition around 50%.

#### 4.9.2. Azion-Bis 3-Ethylbenzothizoline-6-Sulfonic Acid (ABTS) Activity

The ABTS assay was performed following the method outlined by Re et al. [53], slightly modified [54], based on the discolouration kinetics of the 2,2′-Azino-bis (3-ethylbenzothiazoline-6-sulfonic acid (ABTS) ion. ABTS was dissolved in distilled water at a concentration of 7 mM and incubated with an equivalent volume of potassium persulfate solution (2.45 mM in water) for 12 to 16 h in darkness at room temperature. Before use, the ABTS solution was diluted with water to absorb 0.700 ± 0.02 at 734 nm. In each microplate well, 160 μL of ABTS solution was mixed with 40 μL of extract or reference (BHT and BHA) at different concentrations (0–800 μg/mL). Absorbance readings were taken at 734 nm after a 10 min incubation in darkness at room temperature. Three replicates were performed for each sample or standard concentration. The percentage of inhibition was calculated using the following formula:

I% = [(Abs_s_ − Abs_c_)/Abs_c_)] × 100

where Abs_s_ represents the absorbance of the sample and Abs_c_ is the absorbance of the negative control.

The IC_50_ was calculated from three successive points of percentage inhibition around 50%.

#### 4.9.3. Phenanthroline Activity

The phenanthroline assay was conducted according to the protocol described by [55]. In microplate wells, 10 μL of extract or standards (BHT and BHA) at varying concentrations (0–200 μg/mL), 50 μL of FeCl_3_ (0.2%), 30 μL of phenanthroline (0.5%), and 110 μL of methanol were combined and incubated in an oven for 20 min at 30 °C. The absorbance of the samples was measured at 510 nm using a microplate reader, with BHT serving as the standard. The phenanthroline assay result was expressed as A0.5, the sample concentration required to yield a 0.5 absorbance.

#### 4.9.4. Total Antioxidant Capacity (Phosphomolybdenum Test)

The total antioxidant capacity (TAC) was determined using the phosphomolybdenum method described by [56]. A 0.4 mL aliquot of appropriately diluted sample extract was mixed with 4 mL of the reagent (0.6 M sulfuric acid, 28 mM phosphate buffer, and 4 mM ammonium molybdate) in a test tube. The tubes were then incubated at 95 °C in a water bath for 90 min. After cooling, the absorbance was measured at 695 nm. Ascorbic acid was used as the reference compound and tested under the same conditions. Three tests were performed for each experiment. The total antioxidant capacity was expressed as A0.5, representing the sample concentration required to yield 0.5 absorbance.

### 4.10. Inhibition of Enzymes Activities

#### 4.10.1. Alpha-Amylase Inhibition Activity

The inhibitory effect of the extracts of both algae, *J. rubens* and *S. muticum*, on α-amylase enzyme were determined by measuring starch hydrolysis with iodine potassium iodide as a coloring reagent, as described by [59]. The absorbance was measured spectrophotometrically at 405 nm. Acarbose was used as a positive control. The results were expressed as 50% inhibition concentration (IC_50_).

#### 4.10.2. Urease Inhibition Activity

Urease inhibitory activity was determined by measuring ammonia production using the indophenol method as described by Weatherburn [60,61]. Briefly, a reaction mixture containing 25 μL of enzyme solution (Jack bean urease), 10 μL of the tested compound, and 50 μL of urea solution was incubated at 30 °C for 15 min in a 96-well plate. 45 μL of phenol reagent and 70 μL of alkali reagent were added to each well. After 50 min of incubation, the absorbance was measured at 630 nm using a 96-well microplate reader (PerkinElmer, EnSpire Multimode Plate Reader, Cridersville, OH, USA, USA). Thiourea was used as a standard inhibitor. The results were given as a percentage inhibition (%) of the enzyme at the tested compound’s 200 μg/mL concentration. The urease inhibition activity was calculated using the following equation:

Urease inhibition % = (A_control_ − A_simple_)/(A_control_) × 100

### 4.11. Statistical Investigation

The results were expressed as mean values with standard deviation (mean ± SD) based on three measurements. IC_50_ and A0.50 values were calculated using linear regression analysis, and variance was analyzed through ANOVA using XL STAT (2016.02.2845, Addinsoft, Bordeaux, France). Differences between means were evaluated using the Tukey test, with statistical significance at *p* < 0.001.

## 5. Conclusions

This study highlights the efficacy of advanced methodologies for extraction, particularly UAE and MAE, in isolating phenolic substances from macroalgae, specifically red *J. rubens* and brown *S. muticum*. The results showed a high concentration of phenolic compounds, particularly flavonoids, in these species, which correlate with the strong antioxidant properties revealed by four different assays: DPPH, ABTS, total antioxidant capacity, and the phenanthroline assay. The inhibition of enzymatic activity, specifically α-amylase and urease, demonstrated significant results for *S. muticum* in comparison to reference compounds, underscoring the potential of these extracts as bioactive agents predominantly comprising polyphenols. These findings are increasingly relevant to the scientific community investigating the use of biological molecules to treat a wide range of diseases, often with greater efficacity and fewer side effects compared to synthetic alternatives. This study illustrates the benefits of harnessing the rich bioactive compounds found in macroalgae, which show noteworthy biological activities. It is essential to meticulously choose extraction processes, as they directly affect the quality and efficacy of the extracted compounds. These factors necessitate additional examination and advancement.

## Figures and Tables

**Table 1:** The yield of *J. rubens* and *S. muticum* extracts.

Extracts	Yield %
MAE-Jr	6.97 ± 0.10 ^c^
UAE-Jr	6.84 ± 0.60 ^d^
MAE-Sm	23.66 ± 0.10 ^b^
UAE-Sm	24.84 ± 0.11 ^a^

All values are presented as mean ± standard deviation. Values with different superscripts (a, b, c, d) in the same column are significantly different (*p* < 0.001). MAE-Jr refers to microwave-assisted extraction of *J. rubens*, while UAE-Jr denotes ultrasound-assisted extraction of *J. rubens*. MAE-SM indicates microwave-assisted extraction of *S. muticum*, and UAE-SM refers to ultrasound-assisted extraction of *S. muticum*.

**Table 2:** The bioactive components profile of *J. rubens* as determined by UPLC-ESI-MS-MS. ND *=* not detected; +/−: positive/negative; Rt: retention time; ESI: electrospray ionization; Voltage CE: capillary electrospray.

Name	Molecular Formula	Molecular Weight (g·mol^−1^)	Precursor Ion *m*/*z*	Product Ion *m*/*z*	ESI (+/−)	Voltage CE (v)	Rt (min)	Max Intensity	Area %	Rt (min)	Max Intensity	Area %
							MAE *J. rubens*			MAE *J. rubens*		
Epicatechin	C_15_H_14_O_6_	290	290.8000	139.1500	+	−15	4.494	11,737	0.36	5.934	44,561	0.51
Quercetine	C_15_H_10_O_7_	302	303.0500	153.1000	+	−52	6.326	175,097	3.39	6.123	179,517	0.96
Resveratol	C_14_H_12_O_3_	228	229.0500	107.1000	+	−15	4.362	117,205	**12.26**	4.370	122,152	9.52
Luteolin	C_15_H_10_O_6_	286	286.7500	153.0000	+	−32	5.647	13,775	1.77	5.814	−4207	0.03
Naringenin	C_15_H_10_O_6_	286	273.0500	153.0000	+	−23	6.732	28,732	1.22	6.898	64,012	1.67
Oleuropein	C_25_H_32_O_13_	540	540.5000	88.9500	−	−30	6.100	96,468	4.41	6.105	890,146	**49.27**
Riboflavin	C_17_H_20_N_4_O_6_	376	377.1000	360.3000	+	−7	6.463	8,392,792	**70.58**	6.461	5,883,924	**31.95**
Curcumin	C_21_H_20_O_6_	368	368.9000	177.0500	+	−20	ND	ND	ND	5.127	71,491	0.62
Rutin	C_27_H_30_O_16_	610	611.0000	303.1000	+	−21	4.227	32,328	2.17	4.251	30,882	2.07
Beta Carotene	C_40_H_56_	536	537.1000	280.9500	+	−23	5.749	25,247	1.77	6.171	52,511	0.75
2-Meythoxybenzoic Acid	C_8_H_8_O_3_	152	153.0500	135.0000	+	−14	6.110	10,691	1.35	5.412	77,289	1.09
Kojicacid	C_6_H_6_O_4_	142	142.7500	40.9500	+	−19	ND	ND	ND	4.097	2246	0.14
Oleanolic Acid	C_30_H_48_O_3_	456	457.3000	411.4000	+	−15	6.222	16,388	0.69	6.196	28,516	1.35
Sinapic Acid	C_11_H_12_O_5_	224	225.0000	207.1500	+	−10	5.218	76,244	2.21	ND	ND	ND

Bold indicate the components which have the maximum and minimum of area intensity.

**Table 3:** UPLC-ESI-MS-MS-determined bioactive components profile of *S. muticum*. ND *=* not detected; +/−: positive/negative; Rt: retention time; ESI: electrospray ionization; Voltage CE: capillary electrospray.

PA	Molecular Formula	Molecular Weight (g·mol^−1^)	Precursor Ion *m*/*z*	Product Ion *m*/*z*	ESI (+/−)	Voltage CE (v)	Rt (min)	Max Intensity	Area %	Rt (min)	Max Intensity	Area %
							MAE *S. muticum*			UAE *S. muticum*		
Epicatechin	C_15_H_14_O_6_	290	290.8000	139.1500	+	−15	5.654	48,026	**9.29**	5.470	19,268	2.74
Quercetin	C_15_H_10_O_7_	302	303.0500	153.1000	+	−52	5.576	171,287	2.40	4.925	223,283	1.72
Resveratol	C_14_H_12_O_3_	228	229.0500	107.1000	+	−15	4.183	75,799	**8.88**	4.181	639,005	**52.29**
Luteolin	C_15_H_10_O_6_	286	286.7500	153.0000	+	−32	6.718	12,680	1.69	5.256	−8693	0.11
Naringenin	C_15_H_10_O_6_	286	273.0500	153.0000	+	−23	5.900	25,919	1.12	4.804	42,456	0.37
Oleuropein	C_25_H_32_O_13_	540	540.5000	88.9500	−	−30	6.104	137,985	9.86	6.033	125,455	3.46
Riboflavin	C_17_H_20_N_4_O_6_	376	377.1000	360.3000	+	−7	6.461	6,500,747	**59.11**	6.463	4,478,970	**31.74**
Curcumin	C_21_H_20_O_6_	368	368.9000	177.0500	+	−20	5.144	30,300	0.85	6.375	77,141	0.74
Rutin	C_27_H_30_O_16_	610	611.0000	303.1000	+	−21	4.250	28,700	2.90	4.244	27,139	2.04
Beta Carotene	C_40_H_56_	536	537.1000	280.9500	+	−23	6.397	75,339	2.75	6.393	64,504	3.83
2-Meythoxybenzoic Acid	C_8_H_8_O_3_	152	153.0500	135.0000	+	−14	4.566	1748	0.29	4.988	20,125	0.4
Kojicacid	C_6_H_6_O_4_	142	142.7500	40.9500	+	−19	3.155	15,528	0.45	ND	ND	ND
Oleanolic Acid	C_30_H_48_O_3_	456	457.3000	411.4000	+	−15	5.823	11,535	0.33	4.068	16,795	0.52
Sinapic Acid	C_11_H_12_O_5_	224	225.0000	207.1500	+	−10	4.248	95,165	2.50	4.247	207,056	6.61

Bold indicate the components which have the maximum and minimum of area intensity.

**Table 4:** Phenolic, flavonoid, and tannin contents of *J. rubens* and *S. muticum*.

Extracts	Total Phenolic (μgGAE/mg)	Total Flavonoids (μgQE/mg)	Total Tannins (μgEqC/mg)
MAE *J. rubens*	71.83 ± 0.96 ^b^	38.04 ± 0.98 ^a^	64.19 ± 0.76 ^c^
UAE *J. rubens*	48.31 ± 0.32 ^d^	31.38 ± 0.73 ^b^	52.65 ± 1.33 ^d^
MAE *S. muticum*	68.5 ± 0.96 ^c^	42.65 ± 0.36 ^b^	107.52± 2.61 ^b^
UAE *S. muticum*	74.42 ± 0.80 ^a^	58.47 ± 0.24 ^a^	133.67 ± 1.60 ^a^

All values are expressed as mean ± standard deviation. (*n* = 3). The values with different superscripts (a, b, c, d) in the same column are significantly different (*p* < 0.001).

**Table 5:** The results of antioxidant activity of *J. Rubens* and *S. muticum*.

	DPPH IC_50_ (μg/mL)	ABTS IC_50_ (μg/mL)	Phenanthroline Assay A0.5 (μg/mL)	TAC Assay A0.5 (μg/mL)
MAE *J. rubens*	57.13 ± 0,75 ^b^	31.38 ± 0.01 ^c^	87.98 ± 0.55 ^b^	192.42 ± 0.69 ^d^
UAE *J. rubens*	53.97 ± 0.39 ^c^	26.58 ± 0.39 ^d^	79.80 ± 0.68 ^c^	249.98 ± 0.51 ^c^
MAE *S. muticum*	87.55 ± 0,69 ^a^	48.18 ± 0.45 ^a^	61.74 ± 0.86 ^d^	404.55 ± 0.79 ^b^
UAE *S. muticum*	32.67 ± 0.43 ^d^	44.56 ± 0.78 ^b^	97.41 ± 0.58 ^a^	730.56 ± 0.96 ^a^
BHT *	3.08 ± 0.09 ^f^	1.73 ± 0.23 ^g^	33.35 ± 0.93 ^e^	150.18 ± 0.37 ^e^
BHA *	2.03 ± 0.01 ^g^	2.09 ± 0.07 ^f^	10.21 ± 0.06 ^g^	18.94 ± 0.38 ^g^
Ascorbic acid *	6.28 ± 0.14 ^e^	3.46 ± 0.06 ^e^	13.93 ± 0.26 ^f^	37.91 ± 0.24 ^f^

*: Reference compounds. IC_50_ and A0.5 values are the concentration of 50% inhibition percentages and the concentration at 0.50 absorbance, respectively. IC_50_ and A0.5 were calculated by linear regression analysis and expressed as the mean ± SD (*n* = 3). The values with different superscripts (a, b, c, d, e, f, g) in the same column are significantly different (*p* < 0.001).

**Table 6:** The results of enzymatic inhibition activity of *J. rubens* and *S. muticum*.

Inhibition Activity	α-Amylase IC_50_ (μg/mL)	Urease IC_50_ (μg/mL)
MAE *J. rubens*	381.497 ± 1.51 ^b^	134.79 ± 1.35 ^a^
UAE *J. rubens*	-	75.24 ± 3.36 ^b^
MAE *S. muticum*	48.12 ± 2.74 ^c^	˂3.125
UAE *S. muticum*	-	˂3.125
Acarbose	3431.01 ± 2.72 ^a^	NT
Thiourea	NT	8.42 ± 0.06 ^c^

NT: not tested, -: no activity. IC_50_ was calculated by linear regression analysis and expressed as the mean ± SD (*n* = 3). The values with different superscripts (a, b, c) in the same column are significantly different (*p* < 0.001).

## Data Availability

The data presented in this study are available for a limited time upon request from the corresponding author.
